# Local Knowledge and Use of Medicinal Plants in a Rural Community in the Agreste of Paraíba, Northeast Brazil

**DOI:** 10.1155/2021/9944357

**Published:** 2021-12-29

**Authors:** Ezequiel da Costa Ferreira, Maria da Glória Vieira Anselmo, Natan Medeiros Guerra, Camilla Marques de Lucena, Cattleya do Monte Pessoa Felix, Rainer W. Bussmann, Narel Y Paniagua-Zambrana, Reinaldo Farias Paiva de Lucena

**Affiliations:** ^1^Postgraduate Program in Development and Environment (PRODEMA), Ethnobiology and Environmental Sciences Laboratory, Federal University of Paraíba, João Pessoa, Paraíba State, Brazil; ^2^Federal University of Paraíba, Master's Degree in Agronomy, Areia, Paraíba State, Brazil; ^3^Rural Federal University of the Semiarid, Doctoral Student, Plant Science, Mossoró, Rio Grande do Norte, Brazil; ^4^Ilia State University, Tbilisi, Georgia; ^5^Federal University of Mato Grosso do Sul, Institute of Biosciences, Ethnobotany Laboratory, Research Group on Multidisciplinary and Socioecological Studies, Campo Grande, Mato Grosso do Sul, Brazil

## Abstract

The use of medicinal plants is an important source of therapeutic resources in rural communities and the wide versatility of some species may attract interest for prospecting studies. The aim of this study was to record and analyze local knowledge and the use of medicinal plants in the rural community of Malícia, municipality of Araçagi, Paraíba State, Northeastern Brazil, applying quantitative methods to calculate the Relative Importance (RI) and the Informant Consensus Factor (ICF). Semistructured interviews were conducted with 46 heads of households. The interviews addressed questions about the used parts of the plants, therapeutic indications, and form of use. Therapeutic indications were classified into categories of body systems. The Relative Importance Index (RI) was calculated to verify the species versatility, and the Informant Consensus Factor (ICF) was calculated to verify the consensus of use among informants regarding the body systems. A total of 111 plant species were recorded, inside 101 genera and 47 families. Fabaceae (16 spp.), Lamiaceae, and Myrtaceae (each one with 7 spp.) were the most representative families. *Mentha arvensis*, *Aloe vera*, and *Myracrodruon urundeuva* had the highest RI. A high consensus of use was observed among the informants for neoplasms, nervous system diseases, and infectious and parasitic diseases. Leaves were the part most cited for medicinal use. Regarding the method of preparation, the decoction and the oral administration route stood out. Neoplasms and respiratory system diseases had the highest ICF values. The results indicate a diversified knowledge of the local pharmacopeia and the need for in-depth studies to corroborate the effectiveness of medicinal plants and to understand the dynamics of local knowledge.

## 1. Introduction

The use of plants for the treatment of diseases is ancient among human populations, considering the primary need of people to maintain basic care for their own health [1].

The concept of traditional medicine can be understood as a set of health practices, knowledge and beliefs, incorporating plants, animals, and minerals, as well as the use of spiritual, manual, and exercise therapies, which can be applied in isolation or in combination, to maintain well-being, treatment, prevention, or diagnosis of diseases [2].

Some authors, when classifying diseases mentioned by people, place categories of diseases not recognized by allopathic medicine, but forming part of the nosological universe of informants, as “cultural diseases” or “beliefs” [3, 4].

Even with the advent of allopathic medicine, the use of medicinal plants is still a valuable resource, especially in developing countries, where access to synthetic medicines is often not feasible due to high prices [5, 6], or by the lack of access to the biomedical health system, which often does not cover rural areas, requiring the use of medicinal plants for primary health care because they are more accessible [7].

Other issues involving the use of medicinal plants are the belief in this type of treatment and the search for drugs that do not present as many side effects as the allopathic ones [6, 8, 9]^.^ Even in cases where the biomedical health system is available, combinations between this and the traditional system for health treatments are common [7].

Ethnobotany and ethnopharmacology have often been used in research for the search for new drugs, often due to the low financial and time investment that these surveys demand. While ethnobotany is concerned with researching and documenting the relationships between people and plants, based on the human perception of plant resources, ethnopharmacology deals with the evaluation of traditional preparations used to treat diseases, seeking to test their efficacy [9–11]. Traditional knowledge about medicinal plants, in contrast to modern medicinal trends, tends to be orally transmitted and easily disappears with ethnobotanical and ethnopharmacological studies thus being a useful and necessary way of keeping this knowledge integrated with modern medicine [12]. A possible explanation for the progressive loss of traditional knowledge could be the fact that the advancement of allopathic medicine, as part of the globalization process, in some places may cause the local populations to become disinterested in traditional medicine, either by the younger generations losing interest in learning or the older ones in transmitting it [7–9, 12, 13].

There is also a trend in studies aiming to subsidize future research in search of new drugs, which may be more effective in the treatment of diseases, and with fewer side effects [9].

Given the context of the need to preserve traditional knowledge about the use of medicinal plants, quite a few ethnobotanical studies have been carried out in Brazil, including some in the state of Paraíba [14–21]. These studies showed, however, a greater emphasis on diseases related to the respiratory and digestive systems and encountered anything from 36 to 140 species, mostly indicating greater diversity in the families Fabaceae and Euphorbiaceae. This lies in agreement with a higher register of these families in floristic and phytosociological surveys carried out in the region [22–24]. These studies are spread over different types of traditional communities in Brazil, e.g., farmer communities [25–27], indigenous populations [28,29], quilombolas [30,31], riparian populations [32], and caiçaras [33].

Works of this genre have also been developed in different areas and communities around the world, as a few diverse examples serve healers in Guinea-Bissau [34], valley communities in the Italian Alps [35], indigenous villages in the Limu Mountains, China [36], or indigenous communities in Mexico [37], and children in a school in Argentina [38].

Different hypotheses have been proposed and tested in ethnobotanical studies with medicinal plants such as utilitarian redundancy, which seeks to explain the resilience of local medical systems [39,40]; the hypothesis of diversification, which proposes explaining the incorporation of exotic species to local pharmacopeias through a possible increase in bioactive compounds that these plants can bring [41]; or the hypothesis of appearance, which seeks to explain how medicinal plants are selected considering their chemical composition or ecological characteristics, classifying them as apparent and not apparent, based on the thought that, from a chemical perspective, herbaceous (or nonwoody) species are considered to be apparent, assuming that they contain a higher concentration of metabolites, whereas shrubs and trees (or woody) are considered as nonapparent [27,42]. Equally, from an ecological perspective, species easily visible due to their size (such as trees and shrubs) or life-cycle characteristics are more apparent, while small herbaceous species are considered not apparent in the initial stages of succession [42–45]. In both cases, it is inferred that species considered to be apparent are those that are more frequently collected and used more recurrently.

Considering the high level of use of medicinal plants in developing countries and the growing interest in the field of herbal medicine, it is also necessary to take a closer look at the conservation of these resources, because many communities are dependent on their availability, and many species of medicinal plants have by now appeared on lists of endangered species, creating a need to find practices for a more sustainable use [46].

From all of these varied perspectives, studies on medicinal plants are of great importance in Brazil, given their great biodiversity, territorial dimension, and progressive loss of traditional knowledge due to the impacts of globalization on traditional populations.

The present study aimed to record and analyze local knowledge and the use of medicinal plants in the rural Malícia community of Araçagi, Paraíba, Northeastern Brazil, seeking to verify the local importance of the species and how widespread is the local knowledge about these species for categories of medicinal use. We started with the following questions. What is the number of species known locally for medicinal use? What is the origin and way of life of the species used? Are there differences between the most cited species and the most versatile? Which medicinal categories have the greatest consensus for local use? What parts of plants are used and how are they prepared for medicinal use?

The following study assumes the following hypotheses: (a) in the study area there is a greater knowledge of exotic plants than of the native ones, both in the number of species and in the diversity of uses; (b) there is a correspondence between the most cited species and the most versatile; (c) there is a greater consensus of use for the medicinal categories with the highest number of citations. A point to be highlighted is that the studied community is located in an ecotone area, in the transition between the Mata Atlântica and the Caatinga, where we found species from both biomes, which makes the region with peculiar characteristics.

## 2. Materials and Methods

### 2.1. Geoenvironmental Characterization

**Figure 1:**
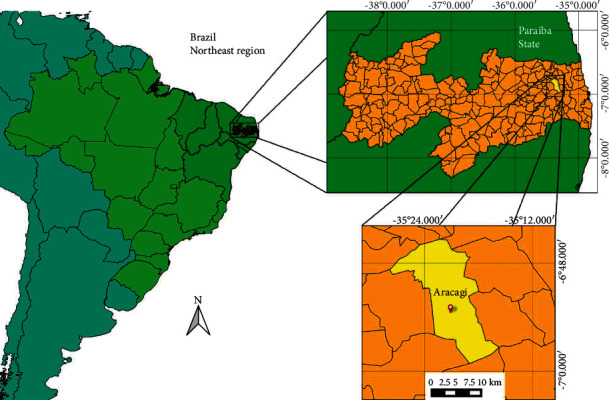
Location of the study area. Municipality of Araçagi, state of Paraíba, the northeastern region of Brazil.

The municipality of Araçagi is located in the mesoregion of the Agreste and microregion of Guarabira, in the state of Paraíba, Northeastern Brazil (Figure 1). It is located between 06°51′10″S and 35°22′51″E [47], with an altitude of 57 m at the municipal headquarters [48]. The region is approximately 64 km from the state capital, João Pessoa, and borders the municipalities of Duas Estradas, Curral de Cima, and Sertãozinho to the north, Mulungú, Sapé, Mari, and Capim to the south, Cuité de Mamanguape, Mamanguape, and Itapororoca to the east, and Guarabira and Pirpirituba to the west. The region has a population of approximately 17224 inhabitants, with 6804 inhabitants in an urban area and 10420 inhabitants in a rural area, with 8574 men and 8650 women. Its territory is 231155 km^2^, with a population density of 74.51 inhabitants per km^2^ [47].

The municipality is part of the geoenvironmental unit of Serrotes, Inselbergs, and Maciços residuais. The vegetation is Caatinga, with small areas of Deciduous Forest. The climate regime is hot, with winter rains from February to August and average annual precipitation around 750 mm [48].

The community of Malícia is about 9 km from the municipal urban center and has a football field, a market, and some small points of commerce.

Access to primary education and basic health is only available in the neighboring community (Agrovila Mulunguzinho), which has a Primary School I (Municipal School of Primary Education João Dutra de Araújo) and a Basic Health Unit, where the residents have access to medical and dental care.

The economy is based on small trading and subsistence farming, with special emphasis on the cultivation of beans, maize, fava, yam, cassava, and the raising of cattle, goats, sheep, and pigs. Some community residents work on a nearby farm, others as day laborers on pineapple plantations around the community. Most of the native forest areas close to the community have been deforested for agricultural practice, more intensely in the last decade, due to the diffusion of pineapple monoculture in the region.

### 2.2. Collection of Ethnobotanical Data

Interviews were conducted with household heads (men and women) [49]. Three visits were made throughout the community between June and August 2015, with the aim of interviewing 100% of the informants. Ultimately 46 people, of whom 17 were men and 29 were women, participated in the interviews.

Before the interview with each person, the research objectives were clearly explained, and each participant was invited to sign the free and informed consent form, requested by the National Health Council, through the Research Ethics Committee (Resolution No. 196/1996) of the University Hospital Lauro Wanderley (CEP/HULW) of the University Federal of Paraíba.

The data collection form involved questions regarding medicinal plants, their used parts, method of preparation, route of administration, disease treated, and socioeconomic information such as age, profession, marital status, and level of schooling of the participants. The names of the species cited were recorded according to the pronunciation of the informants.

Species available with fertile material in local vegetation were collected and herborized in the field and then identified and incorporated in the Jaime Coelho de Moraes Herbarium (EAN), of the Federal University of Paraíba (UFPB), at the Agricultural Sciences Center (CCA).

### 2.3. Data Analysis

Each species was classified according to its place of origin as exotic (from outside Brazil) or native (from Brazil). The plant life forms were classified as herbaceous, shrub, tree, and liana.

The therapeutic indications mentioned by the informants were classified in categories except for cases of insufficient information for such a classification and body systems according to the International Classification of Diseases, version 2015 [50]. Based on this classification, the NBS (number of body systems) and NP (number of properties) were calculated for each species, with the following formula of Bennett and Prance [51]:NBS=NBSS/NBSVS. NBS refers to the number of body systems, resulting from the division of the number of body systems treated by a particular species (NBSS) by the total number of body systems treated by the most versatile species (NBSVS), with species used for a greater diversity of body systems being considered more versatile.

For NP, the following formula was used: NP=NPS/NPVS. Here, NP is the number of properties, resulting from the division of the number of properties attributed to a species (NPS), by the number of properties attributed to the most versatile species (NPVS), with species with the highest number of properties considered more versatile.

The Relative Importance (RI) was then calculated for each species based on the following formula [51]: RI = NBS + NP.

This method emphasizes the species that present greater versatility, i.e., those that present a greater diversity of uses. It represents a quantitative method that does not suffer the direct influence of the number of citations for a certain species, but of the diversity of applications for that species. The maximum value that can be obtained by the calculation is 2; an RI near this value indicates a greater versatility of a species.

The therapeutic properties were grouped into 17 categories: external causes of morbidity and mortality, skin and subcutaneous tissue disorders, ear diseases, diseases of the blood and hematopoietic organs, cardiovascular system diseases, diseases of the digestive system, diseases of the genitourinary system, nervous system disorders, osteomuscular and connective tissue diseases, respiratory system diseases, eye diseases, endocrine, nutritional, and metabolic diseases, infectious and parasitic diseases, injury, poisoning and some other consequences of external causes, neoplasms, symptoms and signs not elsewhere classified, and mental and behavioral disorders [50].

In order to quantify the most important body systems, the Informants Consensus Factor (ICF) was calculated using the formula of Trotter and Logan [52]: ICF=nar − na/nar − 1. Here, nar refers to the sum of the uses mentioned by each informant for a given category; na refers to the number of species cited in the category. The maximum value of the ICF is 1, and the proximity of this value indicates that the informants present a consensus for the category observed.

## 3. Results and Discussion

### 3.1. Identified Plants

A total of 111 plant species were mentioned, of which 1 was identified only at the family level and 109 species were identified at least at the genus level, with 1 undetermined species (Table 1). The species identified are distributed in 101 genera and 47 families. The most significant were Fabaceae (16 spp.), Lamiaceae (7 sp.), and Myrtaceae (7 spp.).

In the state of Piauí, Brazil, only 57 species for medicinal use were observed [53]. This difference may be related to a lower number of informants (31, compared to 46 here interviewed). On the other hand, studies in other parts of the world have recorded a greater number of species, such as Guinea-Bissau (218 sp.) [34], as a consequence of a review of data obtained from healers in different indigenous communities during 17 years, in a riparian community in Brazil (309 sp.) [54] and in a quilombola community in Brazil (133 sp.) [55], which may be related to cultural factors, due to the strong belief in the healing power of plants affirmed by people.

When comparing the results of the present study with those performed in other biomes, we found that, e.g., in the Peruvian Amazon, there was a high representation for Fabaceae with 23 sp., followed by Araceae and Rubiaceae (both with 20 sp.) [56], whereas Lamiaceae and Myrtaceae (both with 4 sp.) were rare. In another Cerrado area, the most important families were Lamiaceae (10 sp.) and Asteraceae (7) [57]. In an area of Atlantic Forest, the highlights were Asteraceae (18 sp.), Lamiaceae (10 sp.), and Myrtaceae (9) [58].

The families Fabaceae, Lamiaceae, and Myrtaceae were also very important in a large study in the state of Piauí, Brazil [53], where Fabaceae presented 8 sp. and Lamiaceae and Myrtaceae showed both 5 sp. In Cariri Paraibano, the predominance of Fabaceae (16 sp.), Asteraceae (11 sp.), and Solanaceae (9 sp.) [59] was observed, and in the semiarid Bahia, Fabaceae (11 sp.), Asteraceae (6 sp.), and Anacardiaceae (5 sp.) [60] were the most important families.

The high number of Asteraceae and Lamiaceae in these studies might be explained by their adaptation to both tropical and temperate environments and by their cosmopolitan distribution [61]. On the other hand, the higher number of Fabaceae in the present study could be explained as maintenance of the knowledge about native species, since only one species of this family was exotic.

### 3.2. Life Form, Origin, and Endemism

There were 43 herbaceous plants, 36 trees, 19 shrubs, and 11 lianas. Of all plants, 56 species were natives of which 44 species were nonendemic natives, 10 endemic natives, and 2 natives without endemic data. Other species were exotic, being 41 cultivated and 12 naturalized (Table 1).

Herbaceous plants were most commonly used, similarly to other studies from Brazil [27, 58, 62] as well as in other countries, such as Iraq [63], Ethiopia [64], Pakistan [65], Ecuador [66], Peru [67], and Bolivia [68]. These results are probably due to the fact that herbaceous plants have greater ease of cultivation or greater availability in areas close to the community. On the other hand, in a different Caatinga area in Brazil, a greater number of tree species were observed [60], similarly to Guinea-Bissau [34], probably due to climatic conditions not favorable to the development of herbaceous species in these areas.

The predominance of herbaceous plants can be explained by the hypothesis of appearance, from the biochemical perspective, which considers herbaceous plants as apparent by their greater production of chemical substances as a defense against herbivory [27].

The use of 36 trees, 19 shrubs, and 11 lianas species can also be considered representative when compared to data obtained from floristic and phytosociological studies in areas of Caatinga which mention 17 to 91 shrub-tree species or woody species [23, 24, 69–71]. Various studies on medicinal plants of the Caatinga have been published, and when only the woody species (shrubs and trees) and native species of this biome are observed, we found a similar number of species, with small variations. The present study recorded 66 medicinal woody species, compared to other studies in this biome that recorded 51 [25], 58 [20], and 37 [72]. This research evidences the vast array of woody species being recognized with medicinal potential in the northeastern semiarid region, requiring studies that more specifically evaluate its potential as a new drug.

A practically equivalent number of native species (56) was observed in relation to the number of exotic species (53). Some studies in Caatinga areas have recorded even a larger number of exotic species [73,74]. In these cases, a high number of exotic species can occur because these plants have greater availability and easier cultivation [73].

The hypothesis of diversification provides a possible explanation for the incorporation of exotic species into local pharmacopeias, considering that this insertion occurs through the need to expand the types of chemical components available for treatment [41].

A relatively high number of species was cultivated, similar to what was found in another semiarid area in Brazil [74]. In the Peruvian Amazon, native species of spontaneous occurrence predominated, which may have occurred due to the wide plant diversity and medicinal knowledge recorded in the study (303 species, of which 199 were native) [56]. The large number of cultivated species observed in the present study can be related to the herbaceous life form which makes cultivation easier in yards. Such life form also stood out in this research.

Another factor observed in the studied community that may be related to the predominance of cultivated species was the fact that there were almost no more native forest areas left close to the community since most of the area was converted into areas for agriculture and pasture. Other authors also present the urbanization process as responsible for the loss of forest areas and the consequent decrease or local extinction of native species [75]. Thus, medicinal plants grown in backyards, which are mostly herbaceous and exotic species, were an easier and viable way to obtain medicinal resources.

### 3.3. Plant Parts, Methods of Preparation, Routes of Administration, and Therapeutic Indications

**Figure 2:**
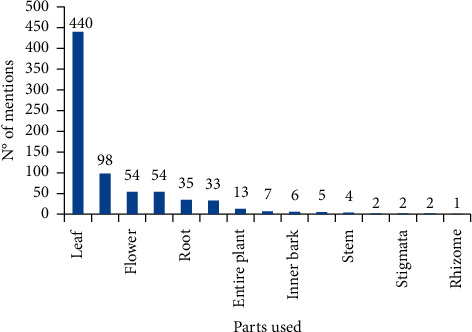
Mentions of plant parts known for medicinal use by residents of the Malícia community, Araçagi-PB (Northeastern Brazil).

Leaf was the most frequently used plant part (440 citations), bark (98), and flower and fruit (54 each) (Figure 2). Similar results were found in the state of Paraná, Brazil [62], Peru [56], Bolivia [68], and Madagascar [76]. In the semiarid region of Paraíba, Brazil, a greater use for bark was observed [25]. In Mexico [77], as observed among healers in Peru [78], the use of leaves and the whole plant was more common. The use of a given plant structure may vary depending on availability and user needs [79]. The high leaf use can be explained by the registration of a large number of herbaceous plants, being easier to collect, and there are many traditional medicines prepared with leaves, as well as the fact that the leaf has a higher concentration of metabolites [79]; on the other hand, the bark usually has a greater representation in studies developed in drier areas, where the leaves are not as available [80].

**Figure 3:**
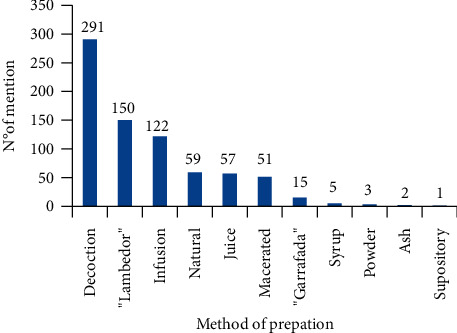
Mentions of methods of preparation of medicinal plants known by the residents of the Malícia community, Araçagi, Paraíba (Northeastern Brazil).

Regarding the preparation mode, there was an emphasis on decoction (291 citations), “Lambedor” (150), and infusion (122) (Figure 3). Decoction and infusion were also highlighted in another area of Agreste in the state of Paraíba, Brazil [17], as well as in Bolivia [68]. “Lambedor” is a local name attributed to a form of medicinal preparation, consisting of the combination and cooking of different barks and/or herbs, forming a kind of syrup, whereas “garrafada” is the maceration of barks, roots, or seeds (together) of different species, in water, cachaça, or white wine.

In the communities of the Italian Alps, the common preparations were infusion, maceration, and cataplasm [35], and in China, crushed material, decoction, and toasting [36].

The oral application was most common (662 citations) followed by topical (82) and inhalation (12). The most common route of administration was oral, which can also be observed in other studies [3, 66, 67]. The oral route may have a faster absorption of the chemicals compared to the other topical routes (topical and inhalation), and it is possible to observe that the topical route has an application more related to the lesions of the skin, whereas the inhalation route is more related to the respiratory system.

The most cited therapeutic indications were flu (116), cough (97), and dyspepsia (indigestion) (48), similar to the findings observed by other authors [8, 81, 82]. The prominence observed for these diseases is possible because these diseases affect the community in a more common way, being more frequent and widespread for the treatments for these diseases.

### 3.4. Most Mentioned and Most Versatile Species

The plants mentioned by the largest number of participants were *D. ambrosioides* (mentruz) (59 citations), *C. citratus* (capim santo) (55), and *L. alba* (51) (erva cidreira), which are all considered exotic in the semiarid region of Brazil, being more prominent for diseases of the respiratory and digestive systems, as well as parasitic diseases, in the case of *D*. *ambrosioides*.

*D. ambrosioides* is a species widely used in folk medicine and described in several studies on medicinal plants with different uses [83], and its antioxidant and anti-inflammatory bioactivity has been observed [84]. The uses mentioned for this species in the present work refer mainly to the treatment of parasitic diseases and the respiratory and digestive systems. The antioxidant bioactivity [84] of this plant might explain its efficacy in the treatment of gastric ulcers, one of the properties attributed to it by informants.

Pharmacological investigations for *C*. *citratus* found several bioactive effects of this plant [85]. Among these effects, we can highlight the antidiarrheal activity and sedative activity [85], which underline some of the indications given to this plant as diarrhea and nervousness.

*L*. *alba* was found to have antiviral and analgesic activity, which might explain the traditional uses indicated for this plant as influenza [86].

It was observed that 10 species presented high versatility with RI ≥ 1. The species with greater versatility were *M*. *arvensis* (Small mint) (RI = 1.71), *A*. *vera* (Babosa) (1.62), and *M*. *urundeuva* (Aroeira) (1.52). In Caatinga areas, the highest values of RI were attributed to *M*. *urundeuva* and *S*. *obtusifolium* [25], similar to other areas of Caatinga in the state of Paraíba [87,88]. In different areas of Caatinga, in the Northeast of Brazil, *M*. *urundeuva* (RI = 2), *A*. *cearensis* (2), and *M*. *rigida* (1,9) [89] were the most versatile.

No correspondence was observed between the most cited species and the most versatile species, indicating that a larger number of citations will not necessarily imply greater versatility (diversity of uses) [51]. Although a species presents a high number of citations, its RI will not be high if these citations are concentrated in restricted groups of properties and bodily systems; in order to obtain a high RI, it is necessary to observe a great diversity of uses attributed to a species.

### 3.5. Categories and Informant Consensus

**Figure 4:**
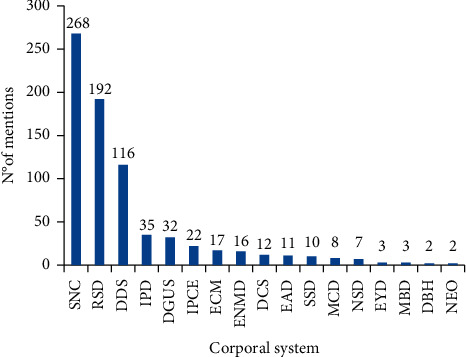
Body system mentions. ECM = external causes of morbidity and mortality; SSD = skin and subcutaneous tissue disorders; EAD = ear diseases; DBH = diseases of the blood and hematopoietic organs; DSC = diseases of the cardiovascular system; DDS = diseases of the digestive system; DGUS = diseases of the genito-urinary system; NSD = nervous system disorders; MCD = musculoskeletal and connective tissue diseases; RSD = respiratory system diseases; EYD = eye diseases; ENMD = endocrine, nutritional and metabolic diseases; IPD = infectious and parasitic diseases; IPCE = injuries, poisoning and some other consequences of external causes; NEO = neoplasms; SNC = symptoms and signs not elsewhere classified; MBD = mental and behavioral disorders.

The most relevant uses were symptoms and signs not classified elsewhere (268 citations), diseases of the respiratory system (192), and diseases of the digestive system (116) (Figure 4). It is common that diseases of the respiratory and digestive systems are frequently treated by traditional medicine [4] as a result of covering the most common diseases that affect the population [90]. However, some differences could be observed in other studies, e.g., the prevalence of digestive system, reproductive system, and cardiovascular system disorders [91], and in the case of emphasis on the treatment of conditions of the skin and gastrointestinal and urogenital systems [92]. We believe that this may be a reflection of the environmental, cultural, economic, and social conditions of the studied regions.

Some studies suggest that the widespread use of medicinal plants to treat diseases of the respiratory system and digestive system may be due to local conditions such as air pollution, presence of impurities in water, and smoke caused by wood-burning, which make the population more prone to manifest these diseases [93]. For example, in the Araguaia microregion in the state of Mato Grosso, Brazil, a higher number of citations were observed for infectious and parasitic diseases, followed by digestive and respiratory system diseases, which may be due to the sanitation conditions recorded in the study [54].

The Informant Consensus Factor (ICF) identified a strong consensus (>0.50) for seven categories, with a greater emphasis on neoplasms (ICF = 1), nervous system diseases (0,83), and infectious and parasitic diseases (0,82) (Table 2). In a region of Mexico, a higher ICF was observed for diseases of the respiratory system (0.92), diseases of the digestive system (0.91), and infections and parasitic diseases (0.89) [77], compared, e.g., to the Italian Alps respiratory (0.88), digestive (0.86), and integumentary (0.83) [35]. It does not seem to be common for the category of neoplasms to present such a high ICF; the explanation for this might be given by the fact that there was only one plant mentioned in two citations for this category.

## 4. Conclusions

The data obtained indicate that the inhabitants of the Malícia community have a broad knowledge of a great diversity of medicinal plants, with different therapeutic applications.

The slightly higher use of native species than exotic species shows that although there has been a significant loss of native forest in the region, the local population maintains the practice and the knowledge of native plants, growing or even buying, in some cases, native plants unavailable in the region. However, a high number of uses of exotic species were also recorded, which may indicate a possibility of proving the hypothesis of diversification, that is, to analyze if the introduction of exotic species into the local pharmacopeia occurs as a possibility of treatment of a wider set of diseases, and it would be worth to investigate what other factors may be interfering with the dynamics of local knowledge. This might lead to reduced pressure on native species. It would also be interesting to conduct further studies in pharmacology and toxicology to confirm plant efficacy, as well as to identify if there is any toxicity in the mentioned plants.

## Figures and Tables

**Table 1:** Data on medicinal plants: life forms, origin and endemism, parts used, and form of use, medicinal uses, number of citations, and Relative Importance.

Family/Scientific name/Voucher	Local name/English name	Life form	Origin and endemism	Parts used	Form of use	Uses	NC	RI
Adoxaceae								
*Sambucus australis* Cham. & Schltdl.	Sabugueira	S	NEN	Fl/Lf	Dc/If/Lb	Headache/Body aches/Fever/Flu/Cough	17	0.52
Amaranthaceae								
*Alternanthera brasiliana* (L.) Kuntze	Anador	H	NEN	Fl	If/Dc	Headache/Fever	2	0.24
*Beta vulgaris* L.	Beterraba/Beet	H	EC	Ro	Lb	Flu	1	0.19
*Dysphania ambrosioides* (L.) Mosyakin & Clemants (25.583)	Mentruz	H	ENT	Lf/Ei	Dc/Nt/If/Lb/Jc	Blood thinning/Indigestion/Bronchitis/Tummy ache/Headache/Stomach/Expectoration/Fever/Flu/Cough/Ulcer/Verminosis	59	1.29
Amaryllidaceae								
*Allium cepa* L. (25.558)	Cebola/Onion	H	EC	Bb/Ei	If/Sy/Mc	Indigestion/Flu/Hoarseness/Cough/Verminosis/Stroke	8	1
*Allium sativum* L.	Alho/Garlic	H	EC	Bl	Nt/Dc	Snake bite/Flu	2	0.38
Anacardiaceae								
*Anacardium occidentale* L. (25.577)	Cajueiro/cashew tree	T	NEN	Ba/Ib/Fr/Sd	Dc/Mc/Jc	Healing/Inflamed tooth/infection/Inflammation/Inside inflammation/Injury/Snake bite/Burn	16	0.95
*Mangifera indica* L.	Manga/Mango	T	EC	Lf	Dc/If	Diabetes/Cholesterol/Malaise	3	0.43
*Myracrodruon urundeuva* Allemão	Aroeira	T	NEN	Ba/Ib/Lf	Dc/Gf/If/Lb/Mc	Healing/Eczema/Expectoration/Infection/Inflammation/Gum inflammation/Uterine inflammation/Urinary inflammation/Injury/Bruise/Cough	27	1.52
Annonaceae								
*Annona muricata* L. (25.593)	Graviola	T	EC	Lf/Sd	Dc	Diabetes/Thrombosis	2	0.38
*Xylopia frutescens* Aubl.	Imbira	T	NEN	Sd	Dc	Tummy ache (abdominal pain)	1	0.19
Apiaceae								
*Daucus carota* L.	Cenoura/Carrot	H	EC	Ro	Jc	Intestinal detoxification	1	0.19
*Eryngium foetidum* L.	Coentro Maranhão/Maranhão cilantro	H	NEN	Lf	Dc	Stroke	1	0.19
*Pimpinella anisum* L. (25.596)	Erva doce/Fennel	H	EC	Sd	Dc/Gf/If	Tranquilizer/Pain/Tummy ache (abdominal pain)/Indigestion/Insomnia/Polycystic ovary	8	0.86
Apocynaceae								
*Catharanthus roseus* (*L*) G. Don (25.579)	Boa noite/Rose periwinkle	H	EC	Fl/Ro	Dc/Nt/If/Lb	Headache/Toothache/Fever/Flu/Cough	11	
	0.67							
*Plumeria rubra* L.	Jasmim vapor/Jasmine vapor	S	EC	Lt	Nt	Injury	1	0.19
Arecaceae								
*Cocus nucifera* L (25.597)	Côco roxo	T	NEN	Lf	Dc	Jaundice	1	0.19
Asparagaceae								
*Aloe vera* (L.) Burm. f (25.553)	Babosa	H	EC	Lf	Dc/Gf/Nt/Lb/Jc/Sp	Cancer/Lumb/Acne/Expectoration/Furuncle/Gastritis/Flu/Hemorrhoids/Inflammation/Skin mark/Prostate/Cough/Ulcer	23	1.62
Asteraceae								
*Acanthospermum hispidum* DC (25.568)	Espinho de cigano/Gypsy-Thorn	H	NEN	Ro	Dc/If/Lb	Inflamed tooth/Flu/Kidney/Cough	4	0.76
*Matricaria chamomilla* (L.) Rauschert	Camomila/Chamomile	H	EC	Fl	Dc/If	Nerves/Tranquilizer	3	0.19
*Helianthus annuus* L.	Girassol/Sunflower	H	EC	Sd	Dc/If	Stroke	2	0.19
Bixaceae								
*Bixa orellana* L.	Açafrão	S	NEN	Sd	Mc	Bruise	2	0.19
Boraginaceae								
*Heliotropium indicum* L. (25.598)	Fedegoso	H	NEN	Ro	Lb	Expectorant/Cough	2	0.38
Brassicaceae								
*Brassica oleracea* L.	Couve/Cauliflower	H	EC	Lf	Jc	Intestinal detoxification	1	0.19
Bromeliaceae								
*Ananas comosus* (L.) Merril (25.560)	Abacaxi/Pineapple	H	EN	Fr	Lb	Bronchitis/Expectoration/Flu/Cough	8	0.48
*Tillandsia recurvata* (L.) L (25.577)	Samambaia	H	NEN	Ei	Dc	Jaundice	1	0.19
Cactaceae								
*Cereus jamacaru* DC.	Cardeiro	T	EN	St	Mc	Kidney stones	1	0.19
*Nopalea cochenillifera* (L.) Salm-Dyck (25.588)	Palma/Forage cactus	S	ENT	St	Mc	Kidney stones	1	0.19
Caricaceae								
*Carica papaya* L.	Mamão/Papaya	S	ENT	Fr	Lb	Flu/Cough	4	0.38
Caryophyllaceae								
*Dianthus caryophyllus* L. (25.563)	Cravo branco/Carnation	H	EC	Fl	Dc	Asthma	1	0.19
Cleomaceae								
*Tarenaya aculeata* (L.) Soares Neto & roalson	Mussambê	H	NEN	Fl/Ro	Dc/Lb	Expectoration/Flu/Cough	9	0.43
Convolvulaceae								
*Ipomoea asarifolia* (Desr.) Roem. & Schult (25.575)	Salsa	H	NEN	Lf/Ei	Dc/Nt	Inflammation/Healing	3	0.38
*Operculina hamiltoni* (G.) Don. F. Austin & Stapies	Batata de purga	L	NEN	Tb	Mc	Lump	1	0.19
Costaceae								
*Costus spicatus* (jacq.) Sw.	Cana do brejo	H	N*∗*	Lf/Ei/Ro	Dc/If	Prostate/Kidney/Urinary Inflammation	5	0.29
Crassulaceae								
*Kalanchoe crenata* (Andrews) Haw (25.585)	Saião	H	ENT	Lf	Dc/Nt/Lb/Jc	Pain/Gastritis/Flu/Cough/Ulcer	17	0.67
Cucurbitaceae								
*Apodanthera congestiflora* Cogn.	Cabeça de nego	L	NEN	Tb	Dc	Itch	1	0.19
*Citrullus lanatus* (Thunb.) Matsum. & Nakai	Melancia/Watermelon	L	EC	Fr/Sd	Dc/Nt	Conjunctivitis/Malaise/Prostate/Urine stimulation	5	0.76
*Cucumis anguria* L.	Maxixe	L	NEN	Fr	Nt	Cough	1	0.19
*Fevillea trilobata* L.	Gindiroba	L	NEN	Sd	Jc	Tiredness/Sinusitis/Constipation	3	0.43
*Luffa operculata* Cong.	Cabacinha	L	NEN	Fr	Dc	Constipation/Lump	2	0.38
*Momordica charantia* L. (25.590)	Melão de São caetano	L	ENT	Lf/Fr	Nt/Jc	Itch/Hemorrhoids/Verminosis	7	0.43
Euphorbiaceae								
*Cnidoscolus urens* (L.) Arthur (25.561)	Urtiga branca	S	NEN	Ro	Dc/Gf/Mc	Expectoration/Infection/Inflammation/Prostate	9	0.62
*Croton jacobinensis* Baill.	Marmeleiro	S	EN	Ba/Lt	Nt/Mc	Healing/Tummy ache (abdominal pain)/Staunch bleeding	3	0.43
*Jatropha mollissima* (Pohl) Baill (25.595)	Pinhão bravo	S	NEN	Lt	Nt	Snake bite	1	0.19
*Manihot esculenta* Crantz	Macaxeira/Manioc	S	NEN	Lf	Nt	Conjunctivitis	1	0.19
*Ricinus communis* L. (25.549)	Carrapateira	S	ENT	St	As	Healing/Eczema	2	0.38
Fabaceae								
*Abarema jupunba* (Willd.) Britton & Killip var. Jupunba	Babatenom	T	NEN	Ba	Gf/Dc/If	Polycystic ovary/Inflammation/Healing	4	0.57
*Amburana cearensis* (Allemão) A. C.Sm.	Cumarú	T	NEN	Ba/Sd	Dc/Gf/Lb/Po	Inflamed tooth/Diabetes/Expectoration/Fever/Flu/Infection/Inside inflammation/Sinusitis/Cough	20	1
*Anadenanthera colubrina* (Vell) Brenan	Angico	T	NEN	Ba	Lb	Stomachache/Expectoration/Flu/Cough	8	0.62
Bauhinia cheilantha (Bong.) Steud	Mororó	T	NEN	Ba	Dc	Sexual impotence	1	0.19
*Bauhinia variegata* L. (25.586)	Pata de vaca	T	EC	Lf	Dc	Hypertension/Diabetes	2	0.38
*Cenostigma pyramidale* (Tul.) E. Gagnon & G. P. Lewis (25.587)	Catingueira	T	EN	Ba/Fl	Dc/If/Mc	Tummy ache (abdominal pain)/Flu/Hemorrhoids/Prostate pain	6	0.76
*Centrosema brasilianum* (L.) Benth	Priquito	L	NEN	Fl	Dc	Flu/Cough	2	0.38
*Erythrina velutina* Willd (25.555)	Mulungú	T	NEN	Ib	Dc	Memory loss	1	0.19
*Hymenaea courbaril* L.	Jatobá	T	NEN	Ba/Ib/Fr	Dc/Gf/Nt/Lb/Mc	Pain/Stomachache/Expectoration/Flu/Hernia/Bruise/Rheumatism	11	1.05
*Libidibia ferrea* (Mart. ex Tul.) L. P.Queiroz var. ferrea (25.565)	Jucá	T	EN	Fr	Dc/Mc	Diabetes/“Open chest” (pain in the sternum region)	2	0.38
*Machaerium hirtum* (Vell.) Stellfeld	Espinho rei	T	NEN	Ba	Dc	Diarrhea	2	0.19
*Mimosa sensitiva* var. malitiosa (Mar.) barneby	Malícia	S	EN	Ro	Dc	Healing	1	0.19
*Pterodon emarginatus* Vogel.	Sucupira branca	T	NEN	Sd	Mc	Backache/Rheumatism	3	0.24
*Senna occidentalis* (L.) Link (25.572)	Mata pasto	S	NEN	Lf	Nt	Lumb	1	0.19
*Vigna unguiculata* (L.) Walp.	Feijão verde/Cowpeas	L	EC	Lf/Fr	Dc/If/Jc	Anticoagulant/Malaise	3	0.38
Fabaceae sp.	Unha de gato	*∗*	*∗*	Lf	Dc	Rheumatism	1	0.19
Geraniaceae								
*Pelargonium graveolens* L'Hér. ex aiton (25.566)	Malva rosa	H	EC	Fl/Lf	Dc/If/Lb	Tammy ache/Fever/Flu/Sinusitis/Cough	13	0.67
Illiciaceae								
*Illicium verum* Hook.f.	Star anise	T	EC	Sd/Fr	If/Dc/Gf	Pain/Polycystic ovary/Infection/Expectoration	5	0.62
Iridaceae								
*Eleutherine bulbosa* (Mill.) Urb.	Alho bravo/Wild garlic	H	NEN	Ei	Dc	Cough/Flu/Expectoration	3	0.38
Lamiaceae								
*Aeollanthus suaveolens* Mart. ex Spreng (25.594)	Macassá	H	EC	Lf/Sd	Dc/Nt/Jc	Stroke/Headache/Earache/Flu/Sinusitis/	10	0.81
*Mentha arvensis* L. (25.578)	Hortelã miúda/Small mint	H	EC	Lf	Dc/If/Lb/Mc/Sc	Amebiasis/Bloated tummy (abdominal fullness)/Bronchitis/Tranquilizer/Stomach cramps/Tummy ache (abdominal pain)/Headache/Nausea/Expectoration/Fever/Gas/Flu/Hemorrhoids/Inflammation/Intestine/Malaise/Gallbladder stone/Kidney stone/Constipation/Cough/Verminosis	46	1.71
*Ocimum basilicum* L.	Alfavaca	H	EC	Lf/Sd	Dc/Nt	Sinusitis/Speck in the eye	2	0.38
*Ocimum gratissimum* L. (25.580)	Louro	S	ENT	Lf	Dc/If	Bloated tummy (abdominal fullness)/Indigestion	5	0.19
*Plectranthus amboinicus* (Lour.) Spreng (25.550)	Hortelã grande/Mint	H	EC	Lf	Dc/If/Nt/Lb/Jc	Lumb/Inflamed tooth/Intestinal detoxification/Tummy ache (abdominal pain)/Headache/Earache/Expectoration/Fever/Flu/Cough	30	1.19
*Plectranthus barbatus* Andrews	Boldo	H	EC	Lf	Dc/If	Bloated tummy (abdominal fullness)/Indigestion/Tummy ache (abdominal pain)	9	0.29
*Rosmarinus officinalis* L.	Alecrim/Rosemary	H	EC	Lf	Dc/If/Mc	Accelerated Heart/Pain/Hypertension/Injury/Malaise/Nervousness	7	0.71
Lauraceae								
*Cinnamomum zeylanicum* Blume	Canela/Cinnamon	T	EC	Ba	If	Indigestion/Pain	2	0.38
*Persea americana* Mill.	Abacate/Avocado	T	ENT	Sd/Lf	Mc/Dc	Injury/Kidney inflammation	2	0.38
Lythraceae								
Punica granatum L.	Romã/Pomegranate	T	EC	Fr	Dc/Mc	Throat/Sore throat/Throat inflammation/Throat infection/Infection/Inflammation/Hoarseness	15	0.67
Malvaceae								
*Abelmoschus esculentus* (L.) Moench (25.569)	Quiabo/Okra	H	EC	Fr	Mc	Diabetes	1	0.19
*Ceiba glaziovii* (Kuntze) K. Schum.	Barriguda	T	EN	Ba	Dc	“Barriga d'água” (Schistosomiasis)	1	0.19
Meliaceae								
*Cedrela odorata* L.	Cedro	T	NEN	Ba	Dc/If	Bloated tummy (abdominal fullness)/Hatched burp (a burp smelling rotten egg)	4	0.19
Moraceae								
*Maclura tinctoria* (L.) D. Don ex Steud	Tatajuba	T	NEN	Lt	Nt	Toothache	1	0.19
Musaceae								
*Musa paradisiaca* L.	Bananeira/Banana tree	H	EC	Fl/Lt	Nt/Lb	Gastritis/Cough	3	0.38
Myrtaceae								
*Eucalyptus* sp.	Eucalipto/Eucalyptus	T	EC	Lf	Dc/Nt/If/Lb/Mc	Tranquilizer/Fever/Throat/Flu/Infection/Sinusitis	18	0.71
*Eugenia uniflora* L (25.591)	Pitanga/Brazilian cherry	S	NEN	Lf	If	Tummy ache (abdominal pain)	1	0.19
*Plinia cauliflora* (Mart.) Kausel	Jabuticaba	T	EN	Lf/Fr/Ba	If/Nt/Mc	Diarrhea/Malaise	3	0.24
*Psidium guajava* L.	Goiaba/Guava	T	ENT	Ba/Lf	Dc/Nt/Mc	Dysentery/Tooth inflammation	7	0.29
*Psidium* sp.	Cumati	S	N*∗*	Lf	Nt	Tummy ache (abdominal pain)	1	0.19
*Syzygium aromaticum* (L.) Nerril	Cravo da India/Indian clove	T	EC	Sd	Dc/Gf	Body pain/Polycystic ovary	2	0.38
*Syzygium cumini* (L.) Skeels	Oliveira	T	ENT	Fl	Dc	Diabetes/Kidney stone	2	0.38
Passifloraceae								
*Passiflora edulis* Sims (25.576)	Maracujá/Passion fruit	L	NEN	Fr	Po	Diabetes	1	0.19
*Turnera subulata* Sm. (25.548)	Nove horas (Nine o'clock)	S	NEN	Fl	If	Cough	1	0.19
Pedaliaceae								
*Sesamum orientale* L.	Gergelim/Sesame	H	EC	Lf/Sd	Nt/Dc	Conjunctivitis/Stroke	2	0.38
Piperaceae								
*Piper nigrum* L.	Pimenta/Pepper	L	EC	Lf	If/Nt	Lumb/Thrombosed hemorrhoids	2	0.38
Phyllanthaceae								
*Phyllanthus niruri* L. (25.552)	Quebra pedra/Stonebreaker	H	NEN	Ei/Ro	Dc/If	Kidney stone/Kidneys	5	0.24
Poaceae								
*Cymbopogon citratus* (DC.) Stapf (25.556)	Capim santo	H	ENT	Lf	Dc/If/Lb	Bloated tummy (abdominal fullness)/Tranquilizer/Tiredness/Pain/Tummy ache (abdominal pain)/Headache/Indigestion/Expectorant/Flu/Fever/Intestine/Nervousness/Blood pressure/Sinusitis/Cough	55	1.19
*Cynodon dactylon* (L.) Pers (25.589)	Grama do rio/River grass	H	ENT	Ei	Dc	Sexual impotence	1	0.19
*Zea mays* L. (25.582)	Milho/Corn	H	EC	Sg	Dc	Swelling	2	0.19
Rhamnaceae								
*Ziziphus joazeiro* Mart.	Juá	T	EN	Ba/Lf	Nt/Lb/Mc	Expectoration/Flu/Seborrhea/Cough	10	0.62
Rubiaceae								
*Borreria verticillata* (L.) G. Mey (25.554)	Vassoura de botão	S	NEN	Ro	Dc/Lb	Lumb/Hemorrhoids/Flu	3	0.57
*Genipa americana* L (25.564)	Genipapo manso	T	NEN	Fr	Mc	Diabetes	2	0.19
*Tocoyena bullata* (Vell.) Mart. (25.581)	Genipapo bravo	S	EN	Ba	Dc/Nt	Bruise/Dislocated finger or toe/Joint pain	3	0.57
Rutaceae								
*Citrus sinensis* (L.) Osbeck (25.551)	Laranja/Orange	T	EC	Fl/Lf	Dc/If/Lb	Bloated tummy (abdominal fullness)/Tranquilizer/Dysentery/Headache/Fever/Flu/Insomnia/Sinusitis/Cough	27	1
*Citrus aurantifolia* Swingle (25.584)	Limão/Lemon	T	EC	Fr	Dc/Jc	Expectoration/Flu/Cough	4	0.43
Ruta graveolens L.	Arruda/Rue	H	EC	Fl	Dc/Nt/If/Mc/Sm	Stomach cramps/Pain/Toothache/Earache/Inflammation	16	0.81
Sapotaceae								
*Sideroxylon obtusifolium* (Roem. e Schult.) Penn.	Quixabeira	T	NEN	Ba	Dc	Bruise	1	0.19
Solanaceae								
*Solanum americanum* Mill (25.592)	Erva moura	H	NEN	Lf	If/Jc	Anticoagulant/Fracture/Injury/Bruise	4	0.43
*Solanum melongena* L.	Beringela/Eggplant	H	EC	Fr	Mc	Cholesterol	2	0.19
Urticaceae								
*Cecropia pachystachya* Trécul	Capeira	T	NEN	Ro	Dc	Backache	1	0.19
Verbenaceae								
*Lippia alba* (Mill.) N. E.Br. ex P. Wilson (25.570)	Erva cidreira	S	NEN	Lf/Ro	Dc/If/Lb	Anemia/“bad tummy” (abdominal pain)/Bronchitis/Tranquilizer/Tiredness/Pain/Tummy ache (abdominal pain)/Headache/Nausea/Expectoration/Fever/Flu/Intestine/Nerves/Cough	51	1.24
Zingiberaceae								
*Alpinia zerumbet* (Pers.) B. L. Burtt & R. M. Sm. (25.559)	Colônia	H	EC	Fl/Lf	Dc/If/Lb	Fever/Flu/Sinusitis/Cough	15	0.48
*Curcuma longa* L.	Açafroa	H	EC	Fl	Dc	Jaundice	1	0.19
*Zingiber officinale* Roscoe.	Gengibre/Ginger	H	EC	Ri	Lb	Bronchitis	1	0.19
Indeterminated								
Indet.	Malva branca	^*∗*^	^*∗*^	Ro	Lb	Flu	1	0.19

H: herbaceous; T: tree; S: shrub; L: liana; EN: endemic native; NEN: nonendemic native; N^*∗*^: native without data found about endemism; ENT: exotic naturalized; EC: exotic cultivated; Ei: entire plant; Bb: bulb; Bl: bulbils; Lf: leaf; Sd: seed; Fr: fruit; Fl: flower; Ba: bark; Ib: inner bark; Lt: latex; Tb: tubercle; St: stem; Ri: rhizome; Ef: stigmata; Ro: root; Sy: syrup; If: infusion; Mc: macerated; In: natural; Dc: decoction; Lb: “Lambedor”; Gf: “garrafada”; Sp: suppository; Jc: juice; As: ash; Po: powder; NC: number of citations; RI–Relative Importance. ^*∗*^Data on life form, origin, or endemism not found.

**Table 2:** Informant consensus factor (ICF) for the categories of medicinal use cited.

Corporal system	No. of species	No. of uses mentioned	ICF
External causes of morbidity and mortality (ECM)	10	17	0,44
Skin and subcutaneous tissue disorders (SSD)	6	10	0,44
Ear diseases (EAD)	3	11	0,80
Diseases of the blood and hematopoietic organs (DBH)	2	2	—
Diseases of the cardiovascular system (DCS)	10	12	0,18
Diseases of the digestive system (DDS)	38	116	0,68
Diseases of the genitourinary system (DGUS)	15	32	0,55
Nervous system disorders (NSD)	2	7	0,83
Musculoskeletal and connective tissue diseases (MCD)	6	8	0,29
Respiratory system diseases (DSR)	41	192	0,79
Eye diseases (EYD)	3	3	—
Endocrine, nutritional and metabolic diseases (ENMD)	14	16	0,13
Infectious and parasitic diseases (IPD)	7	35	0,82
Injuries, poisoning and some other consequences of external causes (IPCE)	16	22	0,29
Neoplasms (NEO)	1	2	1,00
Symptoms and signs not elsewhere classified (SNC)	56	268	0,79
Mental and behavioral disorders (MBD)	3	3	—

## Data Availability

The data used for this study are deposited in the database of the Laboratory of Ethnobiology and Environmental Sciences, of the Federal University of Paraíba, João Pessoa, Brazil, and can be accessed through the previous contact with the authors of this study.
